# Strengths-based positive psychology interventions: a randomized placebo-controlled online trial on long-term effects for a signature strengths- vs. a lesser strengths-intervention

**DOI:** 10.3389/fpsyg.2015.00456

**Published:** 2015-04-22

**Authors:** René T. Proyer, Fabian Gander, Sara Wellenzohn, Willibald Ruch

**Affiliations:** Personality and Assessment, Department of Psychology, University of ZurichZurich, Switzerland

**Keywords:** character, character strength, depression, happiness, online intervention, positive psychology, positive psychology intervention, VIA

## Abstract

Recent years have seen an increasing interest in research in positive psychology interventions. There is broad evidence for their effectiveness in increasing well-being and ameliorating depression. Intentional activities that focus on those character strengths, which are most typical for a person (i.e., signature strengths, SS) and encourage their usage in a new way have been identified as highly effective. The current study aims at comparing an intervention aimed at using SS with one on using individual low scoring (or *lesser*) strengths in a randomized placebo-controlled trial. A total of 375 adults were randomly assigned to one of the two intervention conditions [i.e., using five signature vs. five lesser strengths (LS) in a new way] or a placebo control condition (i.e., early memories). We measured happiness and depressive symptoms at five time points (i.e., pre- and post-test, 1-, 3-, and 6-months follow-ups) and character strengths at pre-test. The main findings are that (1) there were increases in happiness for up to 3 months and decreases in depressive symptoms in the short term in both intervention conditions; (2) participants found working with strengths equally rewarding (enjoyment and benefit) in both conditions; (3) those participants that reported generally higher levels of strengths benefitted more from working on LS rather than SS and those with comparatively lower levels of strengths tended to benefit more from working on SS; and (4) deviations from an average profile derived from a large sample of German-speakers completing the Values-in-Action Inventory of Strengths were associated with greater benefit from the interventions in the SS-condition. We conclude that working on character strengths is effective for increasing happiness and discuss how these interventions could be tailored to the individual for promoting their effectiveness.

## Introduction

One of the applied areas of positive psychology that has generated much interest over the past years is the field of positive psychology interventions (PPIs). The core characteristic of these types of intentional activities is that they are “[…] treatment methods or intentional activities that aim to cultivate positive feelings, behaviors, or cognitions" (Sin and Lyubomirsky, 2009, p. 468). Fordyce (1977, 1983) published two landmark studies on interventions for increasing happiness in students. He proposed 14 fundamentals (e.g., keep busy and be more active, spend more time socializing, develop positive, optimistic thinking, or become involved with meaningful work) that may be linked with happiness. With the emergence of positive psychology the interest in those types of activities has steadily increased and Sin and Lyubomirsky (2009) already list 51 intervention studies in their meta-analysis. Their research provides evidence for the effectiveness of PPIs and they identify specific conditions (e.g., individual vs. group vs. online training), which have an impact on the effectiveness of the interventions (see also Bolier et al., 2013). A recent study also supports the notion that the way people work with a PPI can predict a substantial portion of variance in life satisfaction (6%) and depression (10%) about 3.5 years after completion of the intervention (Proyer et al., 2015). Hence, there is broad evidence that encourages further research in this area.

The present study deals with one specific variant of PPIs, namely strengths-based interventions. Peterson and Seligman (2004) published a classification of 24 strengths and six universal virtues; the Values-in-Action (VIA)-classification. One of the criteria for the inclusion of a strength in the classification was that it should contribute to individual *fulfillment*. Broad evidence has been collected over the past years from correlational studies (using different methods for the assessment of strengths including peer-reports and a broad variety in the samples studied) that the VIA-strengths are positively associated with different indicators of subjective well-being (see Park et al., 2004; Park and Peterson, 2006a,b; Peterson et al., 2007; Ruch et al., 2007, 2010, 2014a,b; Khumalo et al., 2008; Brdar et al., 2011; Proyer et al., 2011, 2013a; Gander et al., 2012; Güsewell and Ruch, 2012; Littman-Ovadia and Lavy, 2012; Buschor et al., 2013; Martínez-Martí and Ruch, 2014; Azañedo et al., 2014; Berthold and Ruch, 2014). When Peterson and Seligman (2004) introduced the VIA-classification, they argued that strengths are malleable and, therefore, could be used for strengths-based interventions ^1^ targeting well-being. Later it has been argued (see e.g., Park et al., 2004) that primarily those strengths should be targeted in interventions that correlate most with life satisfaction. This received initial support from a study where interventions targeting those five strengths that are most correlated with life satisfaction in a 10-week program led to an increase in life satisfaction, while this was not the case for a group that trained in five low-correlated strengths (in a program of equal length; Proyer et al., 2013b). It should be noted, however, that also those participants that were in the group that trained low-correlated strengths reported a subjective benefit from their participation in the program. Additionally, specific strengths seemed to play an important role—irrespective of whether they were directly targeted in the program or not. For example, those participants (in both groups), which reported an increase in self-regulation over the course of the program also reported greater benefit from the interventions (Proyer et al., 2013b).

When thinking about strengths-based interventions the idea of so-called *signature strengths* (SS) is important. Peterson and Seligman (2004) argue that each person possesses three to seven (out of the 24) character strengths, which characterize the person best. They set up several criteria for SS such as, that people experience a feeling of excitement while displaying the strength, or that the use of the strength is invigorating rather than exhausting. Seligman et al. (2005) report findings from a placebo-controlled self-administered online PPI study where one group of participants was assigned to a SS-intervention (“Using SS in a new way”). Participants were instructed to complete the Values-in-Action Inventory of Strengths (VIA-IS; Peterson et al., 2005), which is a subjective measure of the twenty-four VIA strengths. Upon completion participants were “[…] asked to use one of these top strengths in a new and different way every day for 1 week” (Seligman et al., 2005, p. 416). In comparison with a placebo control (PC) condition (writing about early memories), greater levels of happiness were found at 1 week, 1 month, 3 months, and 6 months after the completion of the intervention, and the same results were found for depression with additional effects immediately at the post-test measure. Seligman et al. (2005) also found that the *identification* of one’s SS alone without further consideration had no effects on the dependent variables (happiness and depression).

In a first replication of the findings for the “Using SS in a new way”-intervention with an identical design, Mongrain and Anselmo-Matthews (2012) found comparable results for happiness (effective for up to 6 months), but did not find any effects on depressive symptoms. A further replication of Seligman et al. (2005) with data from German-speaking participants with some adaptations (i.e., advertising the study as a “train your strengths”- rather than an “increase your happiness”-intervention), but with an identical design found similar effects for happiness (effects for 1, 3, and 6 months) and depression (post-test, 1 month, and 6 months and with lower effect sizes for the 3 months time point; Gander et al., 2013). However, in a recent study, which only included 50–70 year old German-speaking participants and the same instructions and design as in the Gander et al. (2013) study, there were effects for happiness (at all post measures), but for depressive symptoms only for the post-test and the 1 month measure (Proyer et al., 2014). Other studies have found effects for SS-interventions for personal well-being as well as an engaged and pleasurable life (Mitchell et al., 2009), and life satisfaction (Duan et al., 2013; see also Bridges et al., 2012). Furthermore, harmonious passion seems to be a moderator of the effectiveness of the intervention on well-being (Forest et al., 2012), whereas extraversion was identified as a moderator of the interventions’ effects on depressive symptoms (Senf and Liau, 2013). It has also been argued that the identification and cultivation of SS should be a core part of interventions in the field of positive psychotherapy (Seligman et al., 2006) and their usage in clinical training has also been advocated (Fialkov and Haddad, 2012). Overall, there is strong evidence that interventions targeting SS are effective in increasing various indicators of subjective well-being. Findings for depression are mixed, but they point toward a potential contribution for ameliorating levels of depressive symptoms as well.

When conducting the “Using SS in a new way”-intervention, participants complete the VIA-IS and strengths are then rank-ordered according to their means. Participants get feedback on their highest five strengths (based on the mean scores) as the signature or top strengths (Seligman et al., 2005). While these strengths fulfill certain characteristics (Peterson and Seligman, 2004), the question arises on whether strengths that are rank ordered on the bottom according to their means also may be useful in strength-based interventions. At this point it is important to note that the VIA-IS does not measure *weaknesses*, but that those strengths only have comparatively lower expressions, which means that participants indicate that they possess the strength to a relatively lower degree. Hence, one might speak of a person’s *lesser* strengths (LS). As mentioned, however, this should *not* be interpreted as the absence and, of course, also *not* as the opposite of a given strength (see Seligman, 2015).

Research has shown that it is fruitful to work on ones SS, but the question arises whether it may also be effective to work on ones LS. There are two studies, which provide first hints on the potential effectiveness of such an approach. Rust et al. (2009) published a preliminary study involving 76 College students who completed the VIA-IS and were randomly assigned to a group that worked on two of their SS (based on the VIA-IS results, selected out of the five SS), or another group who worked on one strength that was a “relative weakness,” and one SS (in addition, a 32-student no-treatment group was tested) for 12 weeks. The dependent variable was life satisfaction assessed via Diener’s et al. (1985) satisfaction with Life-scale. Rust et al. (2009) did not report differences in the gain of life satisfaction between the two intervention groups. If the two intervention groups were pooled they showed larger gains in life satisfaction than the no-treatment group. The authors acknowledge that this is a preliminary study and, of course, it does not provide strong evidence for or against working with the LS – it only seems as if there were no detrimental effects if one of the LS was involved in the intervention.

In a second study, Haidt (2002) published a report on a comparison of students that completed a “strengths-first” program (working on strengths for two weeks based on the VIA-IS) and a “weakness-first” group (working on low scoring, strengths). Students received a list with 120 suggested activities (three to eight for each strength) and were allowed to select what they wanted to do. After the two weeks, the students switched their group assignment and worked on their strengths or relative weaknesses for another two weeks. A broad range of variables (ten dependent variables) were assessed at pre-test, after two weeks (before switching groups), and after another two weeks. After the first two measurement time points the students in the “strengths-first” program reported greater enjoyment of the activities than the “weakness-first” group. Other effects (e.g., subjective well-being, self-esteem, rating of one’s overall health) were weak or mixed and Haidt (2002) concludes that the notion that it may be better to work on a strength than on a weakness (see Buckingham and Clifton, 2001) was not supported. Of course, both of these studies are preliminary in their nature and do not address a comparison directly, but support the notion that it is fruitful to test the differences between interventions targeting *SS* and *LS* (strengths with comparatively low expressions) in more detail. Based on the reported findings, the question emerges whether being instructed that the selected strengths are the personal SS has an effect in itself. To the best of our knowledge it has not been tested thus far whether interventions where participants are assigned to work with selected strengths (varying whether they work with SS or LS without informing them on whether the selected strengths are their signature or their LS) demonstrate similar effects to those reported for the “Using SS in a new way”-intervention.

### The Present Study

In the present study, we examine whether working on character strengths is beneficial, regardless of the individual rank order of these strengths: i.e., independently of working on one’s *signature* or on one’s *LS*. Participants completing the original “Using your SS in a new way”-intervention (as used by Seligman et al., 2005) were explicitly informed that the assigned strengths are their SS. Using this instruction would not allow for a direct comparison with another group of participants working on their LS, since writing a strictly parallel instruction would be difficult in the sense of potentially demotivating participants from engaging in the intervention. Therefore, we decided to adapt the original instruction for our study in order to provide participants in two experimental conditions (SS vs. LS) with identical instructions (see “Procedure” for the detailed instruction). In short, we assigned our participants randomly to three conditions; (1) the *SS* condition, and (2) the *LS* condition, instructing both groups to work on five selected strengths without indicating that these are their SS or LS, or (3) a *PC* condition.

This study has four main aims. The first main aim is (1) testing whether both types of interventions (SS vs. LS) are effective in increasing happiness and ameliorating depression in comparison with a PC (“early memories”; Seligman et al., 2005). The second main aim is (2) investigating whether working on the SS is more effective than working on the LS even if participants are not explicitly informed that these are their SS. It was expected that both interventions would be effective in increasing happiness. Expectations for depression are in the same line, but not as strong (given mixed findings in earlier studies). Participants in our study completed the Authentic Happiness Index (AHI; Seligman et al., 2005) and the Center for Epidemiologic Studies Depression Scale (CES-D; Radloff, 1977) as measures for happiness and depression, but they also completed single item ratings for their satisfaction with (a) life in general; (b) work; (c) leisure time; (d) social life; and (e) health, since most of the literature generated on character strengths is concerned with happiness or other indicators of subjective well-being on a general level, whereas the well-being or satisfaction with different life domains (SLD) is less frequently studied. In addition, as environmental conditions are rarely included, we were interested in testing whether such environmental issues play a role as well. Therefore, the participants also provided ratings on how they see the environmental conditions in each of these categories, irrespective of how satisfied they feel with them. We do not argue that these ratings are *objective* markers as they are based on subjective ratings. These ratings, however, may help in narrowing the gap in the literature on the potential role of circumstantial factors in PPIs. We will analyze perceived changes in happiness in these five different categories and in the analogous environmental factors. Additionally, the data allows the analysis of a “fit”-index between the ratings for satisfaction and environmental conditions. Given the lack of prior knowledge this is more of an exploratory approach, but we expect that there will be different effects for the five categories covered in this study.

The third main aim is (3) testing whether there is a difference in the enjoyment and in the subjective benefit of the different interventions. Based on previous findings (Haidt, 2002) we expected that participants in the SS condition would report higher levels of enjoyment and subjective benefit than those in the other conditions.

Finally, the fourth main aim is (4) testing a set of moderators that may play a role for the effectiveness of the respective intervention. Since all participants complete the VIA-IS it will be tested whether those participants that ascribe themselves more strengths in general, differ from those that ascribe themselves fewer strengths. This will be operationalized by using a total score out of the VIA-IS (the first unrotated principal component) as an indicator of self-ascribed strengths possession or global “virtuousness.” It must be highlighted that this procedure contradicts one of the basic tenets of the VIA-classification, namely the plural nature of the good character. However, other examples have shown that using such a total score can be useful for *research* purposes. For example, Proyer and Ruch (2009) tested the localization of the fear of being laughed at (gelotophobia) in the VIA-classification. While the analysis of the bivariate correlations between each of the twenty-four strengths and the fear of being laughed at provided detailed information on the pattern of relations, the analysis of the total score allowed for a more straightforward interpretation of the data and showed a clearer picture of an underestimation of virtuousness in gelotophobes. Similarly, we argue that the analysis of one total score for the VIA-IS in this particular case will help for a better understanding of who benefits most from the respective interventions. As one aspect of this research aim, we will assess (4a) whether people who ascribe themselves many character strengths benefit more from the interventions (and vice versa). Overall, one might argue that those who ascribe themselves lower levels of virtuousness might benefit more from working on their SS in order to have some pronounced, high-level strengths, whereas those who ascribe themselves higher virtuousness might benefit more from working on their LS, since they already have some pronounced strengths and there is more “room for improvement” in the LS. However, since there are no other studies available for a comparison, this analysis is of an exploratory nature.

These analyses will be followed-up by further investigations based on the character strengths: We will (4b) test whether higher scores in a single strength are predictive for the effectiveness of the intervention in each of the two conditions. Some authors have also reported higher order strengths-factors for the VIA-IS, i.e., a five-factorial solution (i.e., emotional, interpersonal, intellectual, theological strengths, and strengths of restraint) and a two-factorial solution based on ipsative scores (i.e., strengths of the heart vs. mind, and self- vs. other-directed strengths; see Peterson and Seligman, 2004; Peterson, 2006; Ruch et al., 2010; see also Ruch and Proyer, 2015), we will separately test whether higher expressions in these factors are related to the effectiveness of the intervention. Furthermore, we will also address the questions whether (4c) it is important which strengths are among the signature- or lesser-five strengths of an individual; whether (4d) the number of strengths belonging to a strengths-factor among the signature- or lesser five strengths of an individual are predictive for the effectiveness of the intervention; and (4e) whether the (dis-)similarity of the profile with an average profile in the German VIA-IS is predictive for success in each of the two conditions. Since comparatively few data exist on these potentially moderating variables, the analyses are of rather exploratory nature. The main aim of these analyses is testing the impact of individual expressions in strengths and their composition in more detail than what has been reported earlier. The analysis testing the (dis-)similarity with an average VIA-IS profile, which was derived from a large data set of German-speaking adults (*N* = 1,674) that have completed the VIA-IS (Ruch et al., 2010), will provide information on whether deviations from an average VIA-IS profile in any direction is predictive of success in the respective intervention.

## Materials and Methods

### Participants

**FIGURE 1:**
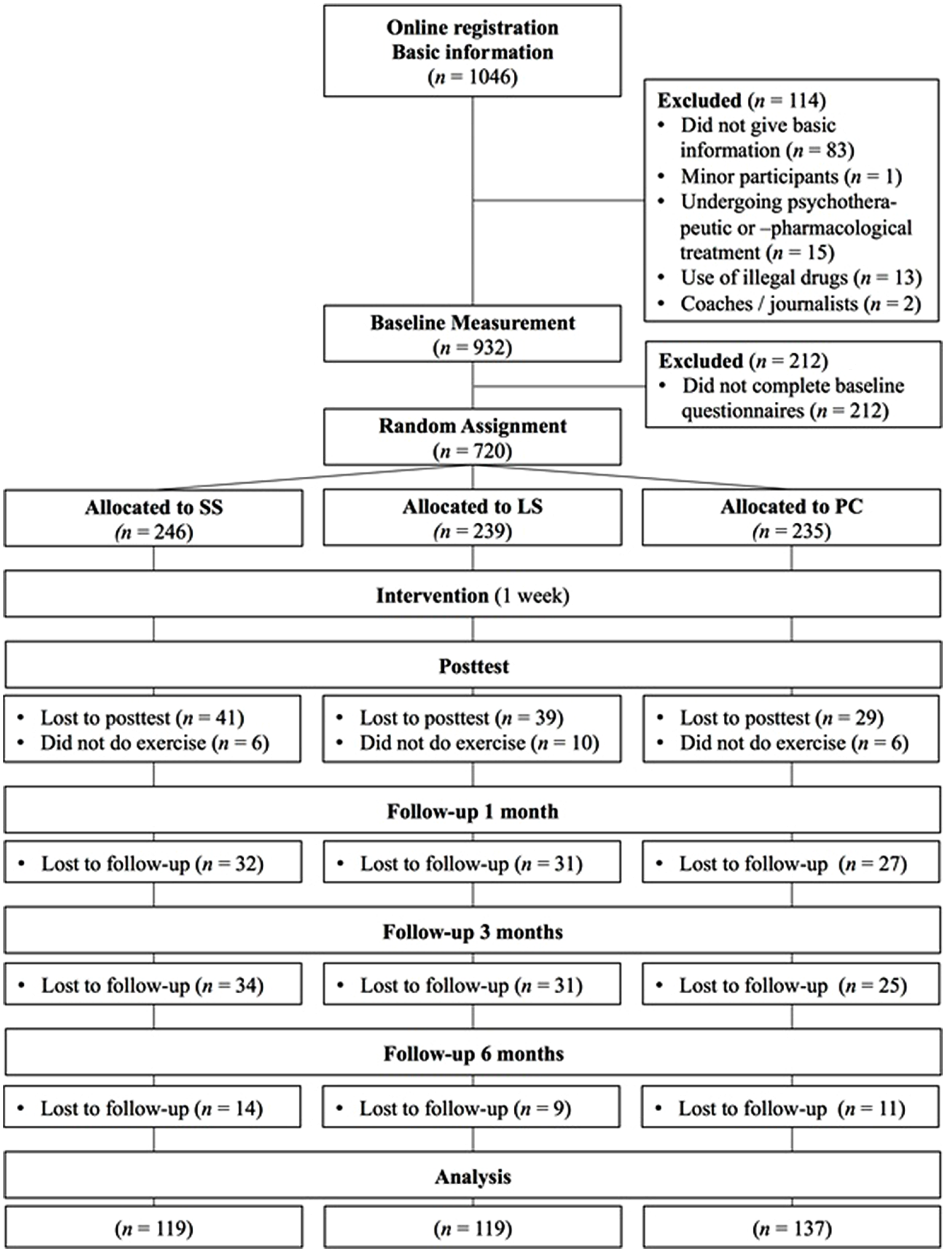
**Flow of participants. SS, signature strengths-intervention; LS, bottom strengths-intervention; PC, placebo control condition**.

A total of 1,046 participants registered on a research website offering a free of charge PPI program. Of these, 720 participants were eligible for participation and were randomly assigned to one of three conditions. Only participants who completed all follow-ups were analyzed (see **Figure 1**). The final sample consisted of *N* = 375 German-speaking adults aged 18 to 77 (*M*= 46.40; SD = 12.31).

Most participants were women (83.7%) of predominantly German (66.7%), Austrian (16.0%), or Swiss (14.9%) nationality. The sample was well educated: more than half (60.5%) held a degree from a university or a university of applied sciences, 20% held a diploma allowing them to attend a university or a university of applied sciences, 16% completed vocational training, and 3.5% completed secondary education. Almost half of the sample was married or in a registered partnership (48.3%), 22.1% were in a partnership (but not married or registered), 16.0% were single, 10.9% were divorced or living in separation, and 2.7% were widowed.

Participants in the three conditions did not differ in their age [*F*(2,372) = 2.55, *p*= 0.08], gender ratio [χ(1, *N* = 375) = 0.78, *p* = 0.68], educational level [χ(6, *N* = 375) = 9.69, *p* = 0.13], or marital status, χ(8, *N* = 375) = 11.80, *p* = 0.15. There were no differences in happiness [*F*(2,372) = 0.60, *p*= 0.55] or depressive symptoms [*F*(2,372) = 0.51, *p*= 0.60] at pre-test.

When analyzing those participants who either did not complete the intervention or did not complete all follow-ups, it was revealed that the latter were on average about 2.6 years older [*t*(718) = 3.00, *p*< 0.01], than those that completed all assignments. They did not differ in terms of the gender ratio [χ(1, *N* = 720) = 1.01, *p* = 0.31], their education [χ(4, *N* = 720) = 1.63, *p* = 0.88], or marital status, χ(4, *N* = 720) = 5.18, *p* = 0.27. Those dropping out were less happy [*t*(718) = 2.92, *p*< 0.01, *d* = 0.15] and reported more depressive symptoms [*t*(718) = -1.98, *p* = 0.05, *d* = 0.22] than those who completed all assignments. Finally, the number of dropouts did not differ across the three groups, *F*(2,717) = 2.76, *p*= 0.06; yet there was a tendency that participants in both strengths conditions dropped out more frequently than in the PC.

### Instruments

The AHI (Seligman et al., 2005; in a German version as used by Ruch et al., 2010) is a subjective measure for the assessment of happiness. It consists of 24 sets of five statements [e.g., ranging from 1 (“I feel like a failure”) to 5 (“I feel I am extraordinarily successful”)] from which one has to choose the statement that describes one’s feelings in the past week best. The AHI was especially designed for monitoring upward changes in happiness (Seligman et al., 2005) and has been often used in PPI studies (e.g., Ruch et al., 2010; Schiffrin and Nelson, 2010; Mongrain and Anselmo-Matthews, 2012; Proyer et al., 2014). In the present study, internal consistency at pretest was high (α = 0.94).

The CES-D (Radloff, 1977; in the German adaptation by Hautzinger and Bailer, 1993) is a 20-item measure for the assessment of the frequency of depressive symptoms in the past week. It uses a 4-point Likert-style scale that ranges from 0 [“Rarely or None of the Time (Less than 1 day)”] to 3 [“Most or all of the time (5–7 days)”]. A sample item is “I felt depressed.” The CES-D has very good psychometric properties and is one of the most frequently used depression measures (Shafer, 2006). In the present study, internal consistency at pretest was high (α = 0.90).

The SLD and conditions in different life domains (CLD) rating forms were developed for this study. They assess the participants’ satisfaction (SLD) and the subjectively estimated quality of the environmental conditions (CLD) in five different life domains; i.e., (a) life in general; (b) work; (c) leisure; (d) social life; and (e) health, with one item each. The scales use a 7-point Likert-style scale ranging from 1 (SLD: very dissatisfied; CLD: very bad) to 7 (SLD: very satisfied; CLD: very good). The ratings were rather stable over the six-month period and ranged in the SLD from *r*_tt_ = 0.50 (social) to 0.61 (work), and in the CLD from *r*_tt_ = 0.49 (work) to 0.58 (health and leisure).

The *Values in Action Inventory of Strengths* (VIA-IS; Peterson et al., 2005; German adaptation by Ruch et al., 2010) is a 240-item measure for the assessment of the 24 character strengths (10 items per strengths) covered by the VIA classification (Peterson and Seligman, 2004). It uses a 5-point Likert-style scale ranging from 1 (very much unlike me) to 5 (very much like me). A sample item is “I find the world a very interesting place” (curiosity). Several studies demonstrated the good psychometric properties of the German version of the VIA-IS (e.g., Proyer and Ruch, 2009; Müller and Ruch, 2011; Güsewell and Ruch, 2012; Buschor et al., 2013; Proyer et al., 2013a; Martínez-Martí and Ruch, 2014). Internal consistencies in the present study ranged from α = 0.71 to 0.92 (median = 0.80).

Besides the scores for the 24 character strengths from the VIA-classification, Peterson and Seligman (2004) identified five higher order factors based on the raw scores (i.e., emotional-, interpersonal-, intellectual-, theological-strengths, and strengths of restraint), whereas Peterson (2006) also reported two higher order factors based on ipsative scores (i.e., strengths of the mind vs. heart, and self- vs. other-directed strengths). Both solutions have also been replicated for the German version of the VIA-IS (Ruch et al., 2010). In the present study, we report analyses for the 24 character strengths, and factor scores for the five- and two-factorial solution of the VIA-IS. Additionally, we also computed a total score of the VIA-IS based on the first unrotated factor of the VIA-IS (see Proyer and Ruch, 2009).

Finally, we also assessed (using single item ratings) how much the participants liked the intervention [from 1 (*not at all*) to 7 (*very much*)], and collected a subjective rating on whether they noticed a personal benefit from the intervention and if so, how they quantify the benefit [from 1 (*no*, *not at all*) to 5 (*yes*, *very high*)].

### Procedure

The study was advertised using online resources (i.e., mailing lists) and media reports. Exclusion criteria were younger age than 18, currently undergoing psychotherapeutic or psychopharmacological treatment, consummation of illegal drugs, and having a professional interest in participation (i.e., being a coach or journalist). The study was conducted online, on a website affiliated with an institute of higher education in the German-speaking part of Switzerland. After registration, participants completed demographic questionnaires, the VIA-IS (see **Figure 1**), the baseline measures of the dependent variables (AHI, CES-D, SLD, CLD), and were randomly assigned to one of three conditions: The SS (or top-) condition, the LS condition, or the PC condition. Participants in both strengths conditions (SS and LS) received feedback on their individual signature or lesser five strengths. ^2^ Further information was provided on strengths in general, with the following training instructions given to the participants in the SS and LS conditions:

“We have selected five character strengths for you. Use one of these strengths in a new and different way every day for 1 week. You can apply the strength in a new environment or when interacting with a ‘new’ person. It is up to you how you want to apply these strengths. Try to apply these strengths, regardless of whether you feel like already using this strength frequently or not.”

Additionally, we added a sentence saying that if participants were unsure on how to implement their strengths on a daily basis, we have provided a list with suggestions in the materials. The list was compiled from Haidt (2002) and Peterson (2006), and other strength-based programs (e.g., Proyer et al., 2013b). Thus, participants in both strengths conditions received identical instructions and differed only in the type of strengths assigned to them (SS or LS). Participants in the PC condition received the “early memories”-exercise (Seligman et al., 2005) and were required to write about an early childhood memory each day for a week. After the intervention week, as well as after 1-, 3-, and 6-months, participants completed measures of the dependent variables. Before each follow-up, participants received reminder emails. Additionally, participants were asked at post-test whether they had completed the assigned intervention. Participants who did not indicate that they had completed the intervention, or failed to complete all follow-ups were excluded from all further analyses. After completion of all assignments, participants received automatically generated, individualized feedback on their character strengths and their level of happiness and depressive symptoms over the course of the full 6 months.

### Data Analysis

We analyzed the effectiveness of the intervention by means of repeated measurement ANCOVAs (repeated measurements = post-test, and follow-ups after 1-, 3-, and 6-months; independent variable = condition; covariate = pre-test score in the dependent variables). Each intervention (SS and LS) was separately compared with the PC condition, and only the main effects for “condition” are reported for an overall assessment of the effectiveness. These analyses were followed by separate ANCOVAs for each measurement time point as a dependent variable (independent variable = condition; covariate = pre-test score). Finally, the two intervention conditions were directly compared with each other, using the same analysis. For facilitating interpretation, the conditions were recoded (0 = PC; 1 = SS and LS), and *t*-scores are reported: Thus, positive *t*-scores indicate that the intervention condition outperformed the PC condition after controlling for the pre-test scores.

For the moderation analyses, we computed the same repeated measurement ANCOVAs for the overall effects as above, with the moderator as an additional continuous independent variable. We report only the interaction between the moderator variable and the condition (but not the main effects for the condition, or the moderator). In a second step, we conducted repeated measurement ANCOVAs for each condition separately to analyze how the moderator affects the outcomes. Again, positive *t-*scores indicate that higher scores in the moderator variables went along with higher expressions in the dependent variables after controlling for the pre-test scores.

## Results

### Intervention Effectiveness: Happiness and Depression

For a first inspection of the trends in the three conditions, mean scores, and SDs for all measurement time points are given in **Table 1**.

**Table 1:** Mean and SD of the three conditions at the five time periods for happiness and depressive symptoms.

		Pre		Post		1 M		3 M		6 M	
	*N*	*M*	SD	*M*	SD	*M*	SD	*M*	SD	*M*	SD
**Happiness**
SS	119	3.01	0.50	3.10	0.52	3.16	0.53	3.13	0.56	3.13	0.58
LS	119	3.05	0.58	3.15	0.56	3.15	0.64	3.21	0.66	3.17	0.67
PC	137	3.09	0.55	3.09	0.57	3.13	0.61	3.11	0.57	3.16	0.58
**Depressive symptoms**
SS	119	0.69	0.46	0.56	0.37	0.61	0.45	0.64	0.44	0.62	0.46
LS	119	0.63	0.46	0.53	0.38	0.59	0.47	0.55	0.45	0.62	0.52
PC	137	0.66	0.45	0.62	0.39	0.62	0.43	0.61	0.43	0.60	0.43

*1 M, one month after the intervention; 3 M, three months after the intervention; 6 M, six months after the intervention; SS, signature strengths-intervention; LS, lesser strengths-intervention; PC, placebo control condition*.

The table shows that happiness increased and depression decreased visually in all conditions, whereas the changes were numerically larger in the intervention conditions than in the PC condition. The results of the repeated measurement ANCOVAs, comparing each intervention separately with the PC condition while controlling for the pre-test scores are given in **Table 2**.

**Table 2:** Overall effects for conditions (intervention condition vs. placebo control condition) and separate analyses for the time periods after the intervention for happiness and depressive symptoms (controlled for pre-test scores in the dependent variables).

	Overall		Post		1 month		3 months		6 months	
	*t*	η^2^	*t*	η^2^	*t*	η^2^	*t*	η^2^	*t*	η^2^
**Happiness**
SS	1.94^∗^	0.02	2.06^∗^	0.02	2.06^∗^	0.02	1.60^†^	0.01	0.67	–
LS	2.04^∗^	0.01	2.71^∗∗^	0.03	1.14	–	2.33^∗^	0.02	0.69	–
**Depressive symptoms**
SS	0.63	–	-2.07^∗^	0.02	-0.46	–	0.19	–	0.14	–
LS	0.82	–	-2.16^∗^	0.02	-0.23	–	-1.13	–	0.63	–

All dfs = 253. η^2^, eta squared; SS, signature strengths-intervention; LS, lesser strengths-intervention.

^†^*p*< 0.10; ^∗^*p* < 0.05; ^∗∗^*p* < 0.01 (one-tailed).

The table shows that there was an overall effect in happiness, but none in depression for both strength conditions. There was an increase in happiness in both conditions at the post-measure as well as at the 3-months time span (marginally significant for the SS condition). The group working with SS also increased in happiness at the 1-month time span. Effects for depression were only found at the post measure, but not at the other follow-ups. A direct comparison of the intervention conditions revealed no differences, neither for the overall effect [happiness: *t*(235) = 0.16, *p*= 0.87; depressive symptoms: *t*(235) = -0.18, *p*= 0.86] nor for the single time points (not shown in detail). Overall, findings were in the expected range and it was shown that both types of interventions had an effect on happiness.

### Intervention Effectiveness: Satisfaction and Conditions in Different Life Domains

We computed the same analyses as reported in the previous section for the single item measures for SLD and the subjective rating of the environmental CLD. Additionally, we tested the “fit” between the two by computing the absolute differences between the standardized variables. Results for SLD, CLD, and their fit are given in **Table 3** (only overall effects for all time points after the interventions jointly are given).

**Table 3:** Effects for condition (intervention condition vs. placebo control condition) on satisfaction with different life domains (SLD), conditions in different life domains (CLD), and their fit (overall time periods after the intervention) controlled for pre-test scores.

	SLD		CLD		Fit	
	*t*	η^2^	*t*	η^2^	*t*	η^2^
**Signature strengths**
General	1.85^∗^	0.01	1.91^∗^	0.02	-1.70^∗^	0.01
Work	1.48^†^	0.01	1.29^†^	0.01	-1.52^†^	0.01
Leisure	1.05	–	1.47^†^	0.01	-1.84^∗^	0.01
Social	0.04	–	-0.12	–	-1.80^∗^	0.01
Health	1.85^∗^	0.01	0.92	–	-0.87	–
**Lesser Strengths**
General	1.11	–	2.15^∗^	0.02	-2.89^∗∗^	0.03
Work	0.38	–	1.33^†^	0.01	-1.18	–
Leisure	0.52	–	0.60	–	-0.87	–
Social	-0.74	–	-0.13	–	-0.64	–
Health	2.01^∗^	0.02	1.00	–	-0.98	–

All values are *t-*scores. All dfs = 1, 248. SLD, satisfaction with different life domains; CLD, conditions in different life domains; Fit, absolute differences of the standardized SLD and CLD; η^2^, eta squared.

*^†^*p*< 0.10; ^∗^*p* < 0.05; ^∗∗^*p* < 0.01 (one-tailed)*.

The table shows that participants in both interventions reported more satisfaction with their health compared to the PC condition. Participants in the SS-intervention also demonstrated effects in the expected direction for the general life satisfaction and showed a trend toward an increase in the satisfaction with work. Regarding the subjective ratings of the environmental conditions, participants in both interventions rated their living conditions in general higher than before, and there was a trend toward rating the work conditions better than before. An inspection of the means (not shown in detail) revealed that the perceived quality of the living conditions was generally rated higher than the satisfaction with them. Scores in satisfaction with life in general increased in both conditions after completion of the intervention, whereas the perceived quality of the living conditions remained more or less stable. In the PC condition, however, satisfaction ratings remained more or less stable, while the quality of the living conditions declined. In the SS condition there also was a trend toward rating the leisure conditions better than before. In both interventions, the “fit” between the ratings of the conditions of life in general and the satisfaction with these conditions *increased* (i.e., the absolute differences between the two decreased) due to the intervention. In the SS condition, the fit also increased for the leisure and social domains, and tended to increase in the work domain.

### Enjoyment and Subjective Benefit of the Interventions

For assessing whether the conditions differed with regard to how much the participants enjoyed (i.e., liked) and perceived a subjective benefit from the interventions, we computed ANOVAs for both variables with the conditions as independent variables. Results revealed that the conditions did not differ in their enjoyment [*F*(2,272) = 0.34, *p*= 0.71] or their subjective benefit, *F*(2,272) = 1.55, *p*= 0.21. However, when comparing both intervention conditions together with the PC condition, a larger subjective benefit was reported for the intervention conditions than for the PC condition, *t*(254) = 1.72, *p*= 0.04, *d* = 0.22.

### Moderating Effects of Character Strengths

In a first step, we were interested in whether the effectiveness of the intervention differed between participants who ascribe themselves generally more (or higher levels of) character strengths and those who ascribe themselves fewer (or lower levels of) strengths. For this purpose, we computed the same repeated measurement ANCOVAs as above, with a total score of character strengths (“virtuousness”) as an additional independent variable. The total score was computed by extracting the first unrotated factor of a principal components analysis.

**Table 4** shows that for those participants in the LS condition, higher scores in virtuousness went along with a stronger reduction of depressive symptoms, when compared with the PC condition. This effect was also present when comparing the LS directly with the SS, *t*(233) = -2.29, *p*= 0.02 (not shown in the table). When analyzing the conditions separately, higher scores in virtuousness were also associated with an increase in happiness for those in the LS condition. When computing a median-split for virtuousness and comparing the effects on happiness between the strengths conditions (SS vs. LS), a significant condition × virtuousness-interaction was found, *t*(233) = 2.52, *p*= 0.01. Simple main effects indicated that for the highly virtuous, the LS condition was more effective [*t*(233) = 1.67, *p* = 0.05; one-tailed], whereas for those low in virtuousness, the SS condition was more effective, *t*(233) = 3.60, *p* = 0.03 (one-tailed test).

**Table 4:** Moderating effects of virtuousness at baseline on happiness and depressive symptoms.

	PC comparison		Separate analyses		
	SS vs. PC	LS vs. PC	SS	LS	PC
*df*	251	251	116	116	134
Happiness	-0.44	1.19	1.07	3.16^∗∗^	1.57
Depression	0.52	-1.97^∗^	-0.73	-3.98^∗∗∗^	-1.76

*All values are *t-*scores. PC comparison = virtuousness × condition (0 = placebo control condition, 1 = signature/lesser strengths-intervention) as predictor of the happiness/depression scores after the intervention (all follow-ups averaged), when controlling for pretest scores in happiness/depression and virtuousness; separate analyses = prediction of happiness/depression scores after the intervention (all follow-ups averaged) by virtuousness when controlling for pretest scores in happiness/depression, for each condition separately; SS, signature strengths-intervention; LS, lesser strengths-intervention; PC, placebo control condition*.

*^∗^*p* < 0.05; ^∗∗^*p* < 0.01; ^∗∗∗^*p* < 0.001 (two-tailed)*.

In a next step, we were interested whether single character strengths, and higher order strengths factors moderate the effectiveness of the intervention. For obtaining the 5-factorial solution (as reported by Peterson and Seligman, 2004 and Ruch et al., 2010) we computed a principal component analysis on the raw scores, extracting five factors (the first seven Eigenvalues were 8.55, 2.54, 1.83, 1.38, 1.10, 1.00, and 0.97, respectively), and rotating them to the VARIMAX-criterion. The factorial solution was similar to the one reported in Ruch et al. (2010; Tucker’s Φ was >0.90 in all cases with the exception for strengths of restraint: Φ = 0.89). For obtaining the 2-factorial solution (as reported by Peterson, 2006 and Ruch et al., 2010), we computed a PCA on ipsative scores (standardized within the participants), extracted two factors (the first four Eigenvalues were 2.92, 2.56, 1.88, and 1.69) and rotated them to the OBLIMIN criterion (delta = 0). The moderating effects of single character strengths, and the higher order strengths factors were tested in repeated measurement ANCOVAs.

**Table 5** shows that participants in the SS condition reported stronger increases in happiness for those that scored higher in love of learning, persistence, and teamwork. Those with higher love of learning reported stronger decreases in depressive symptoms. However, none of these effects reached significance in comparison with the PC condition. Participants in the LS condition reported stronger increases in happiness when they had higher baseline scores in nine out of the 24 strengths, and the higher order strengths factor of interpersonal strengths. For persistence and forgiveness, the moderating effects in the LS exceeded those in the PC. Also, participants in the LS condition reported a decrease in depressive symptoms for those higher in 13 out of the 24 strengths and the strengths factors of emotional and interpersonal strengths. For perspective, persistence, love, kindness, and social intelligence, the effects exceeded those of the PC condition. Finally, those participants in the PC condition who scored higher in curiosity, reported stronger increases in happiness; whereas higher scores in curiosity, zest, self-regulation, hope, and the emotional strengths-factor went along with a stronger reduction in depressive symptoms.

**Table 5:** Moderating effects of character strengths at baseline on happiness and depressive symptoms.

	Happiness					Depressive symptoms				
	PC comparison		Separate analyses			PC comparison		Separate analyses		
	SS	LS	SS	LS	PC	LS	SS	SS	LS	PC
*df*	251	251	116	116	134	251	251	116	116	134
**Character strengths**
Creativity	0.88	0.63	1.39	0.99	0.04	0.88	-0.48	0.71	-1.01	-0.53
Curiosity	-0.65	-0.03	1.07	1.68	2.04^∗^	1.09	0.00	-0.47	-1.87	-2.37^∗^
Open mind	-0.76	-0.52	-0.15	0.18	0.97	0.19	-0.53	-0.28	-1.38	-0.69
Learning	1.02	0.70	2.16^∗^	1.45	0.52	-1.93	-0.93	-2.71^∗∗^	-1.23	-0.02
Perspective	-0.95	0.63	-0.16	1.53	1.02	0.34	-2.15^∗^	-0.45	-3.32^∗∗^	-1.01
Bravery	-0.79	0.26	0.03	1.09	1.05	1.12	-0.62	0.39	-1.59	-1.42
Persistence	1.79	1.99^∗^	2.67^∗∗^	2.76^∗∗^	-0.16	-0.32	-2.04^∗^	-1.32	-3.74^∗∗∗^	-1.58
Honesty	-1.26	0.41	-0.88	1.20	0.75	1.35	-0.82	0.58	-2.24^∗^	-1.38
Zest	-0.81	0.15	0.69	1.85	1.85	1.29	-0.22	-0.41	-2.37^∗^	-3.00^∗∗^
Love	-0.24	1.66	0.88	3.17^∗∗^	0.93	-0.38	-2.35^∗∗^	-1.07	-3.64^∗∗∗^	-0.61
Kindness	0.17	1.73	0.62	2.52^∗^	0.24	-0.40	-2.42^∗∗^	-0.81	-3.56^∗∗^	-0.33
Social I	-1.07	1.10	-0.05	2.67^∗∗^	1.46	0.95	-2.22^∗^	0.11	-4.16^∗∗∗^	-1.41
Teamwork	0.76	0.55	2.05^∗^	1.41	0.70	-0.05	-1.23	-1.13	-2.72^∗∗^	-0.96
Fairness	0.56	1.17	1.47	2.17^∗^	0.83	-0.16	-1.23	-0.98	-2.38^∗^	-0.85
Leadership	0.06	0.08	1.57	1.30	1.37	-0.14	-0.52	-1.56	-1.93	-1.46
Forgiveness	0.27	2.11^∗^	1.09	3.20^∗∗^	0.66	0.22	-1.95	-1.00	-3.54^∗∗^	-1.58
Modesty	1.44	0.94	1.26	0.67	-0.65	-0.57	-1.59	0.16	-1.23	1.01
Prudence	-0.19	0.04	0.08	0.36	0.36	0.06	-1.05	-0.08	-1.59	-0.17
Self-R	-0.93	1.25	0.36	3.20^∗∗^	1.59	0.26	-1.07	-1.69	-3.39^∗∗^	-2.19^∗^
Beauty	1.23	1.10	1.16	0.82	-0.80	-1.09	-1.53	-0.08	-0.68	1.51
Gratitude	-1.05	0.68	-0.07	2.09^∗^	1.35	0.27	-0.55	-0.32	-1.27	-0.87
Hope	-1.74	0.23	-1.31	1.43	1.36	2.53^∗^	-0.43	1.49	-2.36^∗^	-2.81^∗∗^
Humor	-1.69	0.88	-1.62	1.6 9	0.55	1.53	-1.74	1.21	-3.13^∗∗^	-1.06
Religion	-1.11	0.00	-0.29	1.01	1.17	-0.01	-1.10	0.49	-0.89	-0.52
**5-Factor solution**
Emotional	-1.88	0.60	-1.35	1.77	1.40	2.15^∗^	-1.35	0.88	-3.35^∗∗^	-2.74^∗∗^
Interpersonal	0.34	1.11	1.45	2.20^∗^	0.83	-0.30	-1.58	-1.23	-2.99^∗∗^	-0.80
Restraint	0.16	0.46	0.37	0.75	0.20	-0.12	-1.18	-0.38	-1.72	-0.20
Intellectual	1.03	-0.10	1.83	0.21	0.33	-1.12	-0.05	-1.24	0.27	0.33
Transcendence	-0.66	0.80	-0.68	1.19	0.05	0.23	-0.90	0.92	-0.47	0.61
**2-Factor solution**
Heart vs. Mind	0.52	-0.68	0.08	-1.39	-0.51	0.37	0.71	0.52	0.77	0.07
Self vs. Others	0.18	0.32	-0.17	0.19	-0.23	-0.72	0.03	-0.16	0.60	0.91

*All values are *t-*scores. PC comparison = character strength/strength factor × condition (0, placebo control condition; 1, signature/lesser strengths-intervention) and the character strength/strength factor as predictor of the happiness/depression scores after the intervention (all follow-ups averaged), when controlling for pretest scores in happiness/depression and the character strength/strength factor; separate analyses = prediction of happiness/depression scores after the intervention (all follow-ups averaged) by the character strength/strength factor when controlling for pretest scores in happiness/depression, for each condition separately; SS, signature strengths-intervention; LS, lesser strengths-intervention; PC, placebo control condition. Open mind = open mindedness; learning = love of learning; social I, social intelligence; self-R, self-regulation; beauty, appreciation of beauty and excellence; religion, religiousness*.

*^∗^*p*< 0.05; ^∗∗^*p*< 0.01; ^∗∗∗^*p* < 0.001 (two-tailed)*.

We also assessed whether the effectiveness of an intervention depends on *which* character strengths are part of either the signature or the lesser five strengths that people trained (not shown in detail). For this purpose, we compared the effectiveness of the intervention between those participants who had one particular character strength among their SS or LS with those who did not. Results showed that those participants in the strengths condition showed stronger increases in happiness if they had teamwork among their SS, relatively weaker increases if open-mindedness was one of the SS, and stronger reductions in depressive symptoms if love of learning was one of the SS. Those in the LS condition showed weaker increases in happiness when self-regulation was one of the LS. However, it has to be noted, that some strengths were rarely among the SS or LS (groups sizes for the SS ranged from *n* = 6 (zest and self-regulation)] to *n* = 69 (curiosity); for the LS they ranged from *n*= 4 (fairness) to *n*= 61 (modesty)), and the group sizes were, therefore, rather small for some of the comparisons and need to be interpreted conservatively.

Data were not only analyzed at the level of the single strengths, but also for the broader strengths factors. We tested whether there is a difference in the intervention effectiveness for those participants who had more strengths of a specific factor among their SS or LS (not shown in detail). Results showed that in the SS condition, participants reported higher increases in happiness, the fewer strengths of restraint were among their SS. In the LS condition, increases in happiness were stronger for those with more interpersonal strengths among their SS, whereas stronger increases in happiness and amelioration of depressive symptoms were found for those with fewer strengths of restraint among their SS. The effectiveness of the intervention was independent of the number of strengths of a specific factor among the LS.

In a next step, we tested whether intervention effectiveness was also affected by an individual’s profile in the character strengths. More precisely, we examined whether the deviation of an individual’s profile from a profile generated from a large sample of German-speakers that completed the VIA-IS (Ruch et al., 2010) has a moderating effect on happiness and depressive symptoms. For this purpose, we computed the Euclidian distance (i.e., the square root of the sum of the squared differences) between an individual’s profile and Ruch’s et al. (2010) sample. Again, we tested for moderating effects by means of repeated measurement ANCOVAs. Since the overall levels of character strengths might influence the results, we entered the first unrotated factor as an additional covariate (which only led to small changes in the results). Results are given in **Table 6**.

**Table 6:** Moderating effects of the deviation from a normative profile.

	PC comparison		Separate analyses		
	SS	LS	SS	LS	PC
*df*	251	251	116	116	134
*Happiness*	2.03^∗^	-0.50	2.27^∗^	-1.44	-0.55
*Depression*	-0.17	0.76	-0.63	1.18	-0.32

*All values are *t-*scores. The deviation from an average profile was computed as the Euclidian distance between the average scores (as reported by Ruch et al., 2010) and an individual’s strength scores; PC comparison = interaction between condition (0 = placebo control condition, 1 = signature/lesser strengths-intervention) and the deviation from an average profile as predictors of the happiness/depression scores after the intervention (all follow-ups averaged), when controlling for pretest scores in happiness/depression and the deviation from an average profile; separate analyses = prediction of happiness/depression scores after the intervention (all follow-ups averaged) by the deviation from an average profile when controlling for pretest scores in happiness/depression, for each condition separately; SS, signature strengths-intervention; LS, lesser strengths-intervention; PC, placebo control condition*.

*^∗^*p*< 0.05 (two-tailed)*.

The table shows that stronger deviations from the average profile went along with stronger benefits in the SS-interventions, while the deviation did not have an influence on depressive symptoms, and had no effects on the LS condition. Hence, having a somewhat atypical profile (in comparison to those tested by Ruch et al., 2010) was associated with greater benefits for those working on their SS.

## Discussion

The present study extends the knowledge on the effectiveness of strengths-based interventions in several ways. As in earlier studies, an intervention based on one’s SS was effective in increasing happiness. Therefore, the identification and usage of one’s SS in a new way seems to be an effective way to achieve sustainable changes in well-being—even if participants were not explicitly informed that they were working on their SS. This was found for happiness, but also for single-item ratings on different domains of well-being. Hence, this study shows that strengths-based interventions also affect the SLD (i.e., for the SS-intervention the satisfaction with life in general, and the satisfaction with one’s health). Moreover, the SS-intervention was associated with seeing better general living conditions, and it also reduced the discrepancy between the perceived conditions and the satisfaction with them in various life domains (i.e., life in general, and the leisure, and social domains). Of course, we do not know from the current data whether the objective living conditions truly have changed. However, the finding clearly supports the notion that it may be fruitful to study effects not only for happiness in general, but also for specific facets. The latter should not only be tested by self-reports, but also by considering reports from knowledgeable others and other more objective markers.

It is important to acknowledge that findings from our study *cannot* be seen as a replication of the standard “Using SS in a new way”-intervention (see, e.g., Seligman et al., 2005; Mongrain and Anselmo-Matthews, 2012; Gander et al., 2013), but we tested one of its variants. We used the same instruction for the participants in our signature and LS-intervention and only indicated that we selected five strengths for the respective intervention, without further elaborating on the rationale for this selection. When we compared the results with the original instruction, we found that the increases in happiness were observed for a shorter time period (up to 1 month, whereas after 3 months a trend in the expected direction was observed), whereas only short-term reductions in depressive symptoms were found. Of course, our findings warrant further investigation and replication; using the available data we cannot answer the question of whether differences are due to the fact that our participants were not informed that the assigned strengths were their SS, or whether other factors also play a role. It cannot be ruled out, however, that knowing whether the strengths are SS or not might have an effect on *how* participants conduct the intervention (i.e., invest more or less effort), which in turn was previously found to have an impact on the effectiveness of the intervention (e.g., Proyer et al., 2015). One might also argue that differences in the way people work with this intervention in comparison with the original instruction might be small because people will notice whether they “posses” a strength or not (see Peterson and Seligman, 2004), or will have at least an implicit understanding of what their strengths are or not. This, however, needs to be tested further in future studies.

It seemed interesting to us that the findings for the intervention focusing on the LS were in the same direction as the SS-intervention; the intervention led to highly comparable increases in happiness and also increased the satisfaction with one’s health and the perceived quality of general living conditions, and reduced the discrepancy between the perceived quality of the living conditions and the satisfaction with them. Also, the LS-intervention did not differ from the SS-intervention in terms of the enjoyment or perceived benefit. However, we do not argue that the two types of interventions are identical. For example, one might argue that working on SS might be more beneficial for other outcomes, such as fostering *empowerment* (i.e., the perceived meaning, competence, autonomy, and impact; Spreitzer, 1995) than working on LS, since people more easily identify with and work on their SS. Thus, future studies should also consider a broader range of outcome variables.

The finding that we observed an increase in fit between the satisfaction with life in general and the perceived quality of the living conditions in both interventions can mainly be traced back to an increase in the satisfaction ratings. The ratings for the perceived quality of the conditions were rather stable across all time points; in general, the quality of the conditions was rated higher than the satisfaction with them. Thus, this increase in the fit might indicate that due to the intervention the participants were more able to use their potential; i.e., to appreciate the already high quality of living conditions. Of course, this finding needs to be replicated and more objective measures of the living conditions are clearly warranted.

We found strong evidence that character strengths have an impact on the effectiveness of the interventions. For example, if data was split for those high and low in virtuousness based on the VIA-IS (median split in the first unrotated principal component) different patterns emerged for the two groups. In short, for those seeing themselves as virtuous, working with their LS was more effective, while for those that saw themselves as low in their virtuousness, the SS-intervention led to greater effects. Hence, the general level of (self-ascribed) strengths possession may play a role if thinking of increasing the person × intervention-fit (see, e.g., Lyubomirsky et al., 2005; Schueller, 2010; Lyubomirsky and Layous, 2013; Proyer et al., 2015). These findings may also help to understand mixed results in replications of the initial study by Seligman et al. (2005) depending on the strengths outlet of the participants. Thus, pending further replication this finding can have practical implications for tailoring strengths interventions to the participants. As mentioned earlier we do not advocate the interpretation of a general score out of the VIA-IS, but the present analysis has shown that computing such a score for research purposes might facilitate the interpretation of data from strength-based interventions.

For the LS-intervention, a moderating effect was also found at the level of single character strengths; those higher in certain strengths benefited more from the intervention (increases in happiness and reduction in depressive symptoms) than those low in these strengths. For the SS-intervention, only a few strengths moderated the effectiveness of the intervention. The only strength that moderated the effects of both strengths-interventions was *persistence*. This strength may be a good predisposition to continuously work on ones strengths and keeping the focus on the task even when distractions are present. Furthermore, we found smaller effects in the LS condition for those who had self-regulation in their bottom-five strengths. Both strengths are robustly related and show high loadings on the “strengths of restraint”-factor. One might argue that these strengths play an important role in all interventions – not just those examined in the present study. Proyer et al. (2013b) reported similar findings and emphasized the importance of self-regulation in positive interventions. Potentially, the effects of these variables disappear when an intervention is administered in different settings that offer more guidance than the self-administering of interventions as in the present study. This might be important for individuals with low scores in these strengths. Future studies will be needed to test this assumption. Otherwise, we found for both strengths conditions, that they were more effective for those with *fewer* strengths of restraint among their SS. Thus, the findings suggest that a minimum level of these strengths is necessary for an individual to be able to benefit from a self-administered intervention, but scoring too high in these strengths might have detrimental effects on the effectiveness of an intervention.

Finally, we found that those participants whose character strengths-profile differs from a profile derived from a large sample collected for the adaptation of the German-language version of the VIA-IS (Ruch et al., 2010), benefit more from the SS-intervention than those whose profile is closer to a normative one – independently from their overall virtuousness. Thus, this means that the SS-intervention works better for those people who have some strengths that are especially high or low in comparison to the “normative” profile. Of course, the interpretation of this deviation is difficult from a psychological perspective, but it seems as if having a “different” strength profile than the sample of comparison is beneficial for working with strengths. It is important to highlight that our analysis does not allow to say that this refers to single peaks in the sense of *exceeding* the profile in ones strengths—also deviations in the other directions are possible.

At this point it also needs mentioning that the strategy of identifying the SS via the rank order of the mean scores in the VIA-IS is only an approximation and other strategies may be more precise for their identification. For example, Peterson and Seligman (2004) also describe the *Values-in-Action Strengths Interview* (VIA-SI), which was developed for the identification of SS. The usage of the mean scores is a limitation of this study (as it is for other studies applying this strategy for deriving SS). Strengths ranked at sixth or seventh place may numerically be different, but not statistically different from the one ranked at fifth place. If, for example, a participant would say that the strength ranked at position six in the rank-ordered VIA-strengths feels more like a SS to him/her than the one ranked on fifth position, than the effect may be even stronger when working with this strength in the intervention. Therefore, the approximation of using mean scores for the identification of the SS in intervention studies has proven to work well for the purpose of this type of research, but may not be the best possible way of identifying them. Another limitation is the imbalance in the gender distribution in our sample (more women) and their comparatively high educational level. Although there are no reports on major gender-differences in the effectiveness for PPIs, findings should be interpreted conservatively because of this imbalance. We also tested a set of specific moderators, but others (e.g., broader personality variables; see, e.g., Senf and Liau, 2013) may also play a role. Recent research (Ruch and Proyer, 2015) has also suggested that the factor-analytically derived solution we used in this study as mediators may better be replaced with a different solution. Furthermore, it would have been interesting to compare the interventions conducted in this study directly to the original “Using SS in a new way”-intervention to test the differences in more detail.

In line with earlier findings (e.g., Haidt, 2002), we argue that both, working on the SS as well as working on the LS, is beneficial for increasing happiness. However, when also considering the individual character strengths-profile of the participants (such as the overall level of virtuousness), the effectiveness of the intervention might depend on whether one is working on one’s SS or the LS. Therefore, it might be fruitful to take an individual’s character strengths into account when assigning an intervention to a person in order to enhance its effectiveness.

## Acknowledgments

This study has been supported by research grants from the Swiss National Science Foundation (SNSF; grants no. 100014_132512 and no. 100014_149772) awarded to RP and WR. The authors are grateful to Jade Hooper for proofreading the manuscript.

## Footnotes

1 It needs mentioning that we use the term of strengths and strength-based interventions in line with Peterson and Seligman’s (2004) notion of *character* strengths, i.e., morally positively valued traits. This differs from other usages of the word (personal) *strengths* such as, for example, Wood et al. (2011, p. 16), who see strengths as “[…] characteristics that allow a person to perform well or at their personal best […]” and make no references to the moral aspect of *character* strengths.

2 Religiousness was excluded (in both conditions) since we wanted to avoid instructing non-religious individuals to work on their religiousness.
